# Prevalence and Risk Factors of* Helicobacter pylori* Infection among Children Aged 1 to 15 Years at Holy Innocents Children's Hospital, Mbarara, South Western Uganda

**DOI:** 10.1155/2019/9303072

**Published:** 2019-03-07

**Authors:** Phoebe Aitila, Michael Mutyaba, Simon Okeny, Maurice Ndawula Kasule, Rashid Kasule, Frank Ssedyabane, Benson Okongo, Richard Onyuthi Apecu, Enoch Muwanguzi, Caesar Oyet

**Affiliations:** ^1^Mbarara University of Science and Technology, Faculty of Medicine, Department of Medical Laboratory Sciences, P.O. Box 1410, Mbarara, Uganda; ^2^Clarke International University, Institute of Allied Health, P.O. Box 7782, Kampala, Uganda

## Abstract

**Background:**

* Helicobacter pylori* infection affects more than half of the world's population. The infection is generally acquired during childhood but can remain asymptomatic, with long-term clinical sequelae including gastritis, peptic ulcer disease, and stomach cancer.

**Methods:**

The study was approved by Institutional Review Committee of Mbarara University of Science and Technology. After obtaining informed consent from parents/legal guardians, illegible children who presented with gastrointestinal complaints at Holy Innocents Children's Hospital were recruited; structured questionnaires were administered to the parents/guardians to collect information on sociodemographic data and risk factors of* H. pylori* infection. Four (4) millilitres of blood was collected from each child and tested for* H*.* pylori *blood Antibody test and stool specimens were used for* H. pylori* antigen test.

**Results:**

The prevalence of* H. pylori* infection among the study participants was 24.3%. The infection rate increased with increase in age of the participants, from 16.2% among 1to 5 years old to 27.2% among 6 to 10 years. Infections were higher among school going children (68/74, p=0.003, OR 3.9; CI: 1.5 to 10.6) and children from crowded households (59/74, p<0.001, OR 2.6, and CI 1.3 to 5.0), unsafe source of drinking water at schools (46/74, p=0.003), and lack of sanitary facility at homes (57/74, p=0.001, and OR 1.6 CI 0.7 to 3.6).

**Conclusion:**

The prevalence of* H. pylori *infection among children aged 1 to 15 years at Holy Innocents Children's Hospital was high and increases with age. School attendance, lack of sanitary facility, lack of safe drinking water, and overcrowding were the risk factors associated with* H. pylori* infection.

## 1. Background

*Helicobacter pylori* infection is a global public health problem, affecting over 50% of the population worldwide [1, 2]. Infections are thought to occur early in life (during childhood) and the infection implicates several medical conditions including chronic gastritis, gastric cancer, gastric adenocarcinoma, mucosa-associated lymphoid tissue (MALT), lymphoma, and peptic ulcer disease [2–5]. Infected individuals present with gastric reflux, abdominal pain, intestinal bleeding, occasional fevers, and loss of weight which if not treated can result in gastric ulceration and perforation [6]. The incidence and prevalence rates of childhood infection with* H*.* pylori* vary greatly [5]. Within developed nations, prevalence rates of* H*.* pylori* infection among children have been shown to range from as low as 1.8% to as high as 65% [7–13]. While in developing countries the prevalence is generally higher reaching up to 90% in some countries [14–20]. The mode of transmission for* H. pylori* is not certainly known; however, epidemiological studies strongly support person-to-person transmission and fecal-oral and oral-oral routes [21–26]. School going children in developing countries are at higher risk of* H. pylori* infection. Several factors control the transmission in developing countries including low socioeconomic status, poor quality of drinking water, overcrowding, poor personal and environmental hygiene, and food contamination [27–33]. In Uganda, data on the prevalence of* H. pylori* infection in children are scanty. Few studies have been reported in Kampala with an overall prevalence of 43.3% [20]. To the best of our knowledge, there is no available published information on the prevalence of* H. pylori* infection in children in South Western Uganda. The primary objective of this study was to determine the prevalence and risk factors of* H. pylori* infection among children at Holy Innocents Paediatric Hospital in Mbarara South Western Uganda.

## 2. Method and Materials

A cross-sectional study was conducted at Holy Innocents Children's Hospital. The study was approved by Research and Ethics committee of Mbarara University of Science and Technology. Informed consent was obtained from parents or legal guardians of the children before enrolment in the study.

### 2.1. Data Collection

A total of 304 participants aged 1 to 15 years who presented with gastrointestinal complaints were randomly recruited into the study from January 2017 to August 2017. The participants were recruited using simple randomization technique as described previously [34, 35]. Briefly, every child who presented with gastrointestinal complaints had a parent or legal guardian was requested to pick a number written on cards and place it in a box before the recruitment. Every parent or guardian who picked an even number is allowed to consent, and the child is enrolled. The cards were reshuffled each time a card is picked. A simple closed-ended questionnaire was then administered to collect information on age and gender of the participating child, type of home toilet facility, sources of drinking water at home, number of people in their household, number of siblings, family history of peptic ulcers, and the educational level of the parent/guardian. 4mls of venous blood was drawn from each participant into a plain vacutainer tube and processed for* H. pylori* antibodies using rapid antibody-antigen based immunoassay strips (ABON BIOPHARM HANGZHOU CO LTD, CHINA). Stool samples from those that tested positive with antibody test were subjected to* Helicobacter pylori* antigen in human fecal specimen test strip (Vaxpert Inc. suite 355 Two south Biscayne Blvd Miami, Fl, USA). A positive* H. pylori* test was defined as positive antigen test performed on the stool specimen.

### 2.2. Statistical Analysis

The data generated were coded, entered, validated, and analyzed using STATA 12 software (StataCorp, College Station, TX, USA). Associations between categorical variables were tested using the chi-squared test with reports of the corresponding p-values. In some instances where there were small numbers in a given cell (<5), Fischer's exact test was used and the corresponding p-value reported. The odds ratio and the corresponding 95% confidence intervals (95% CI) were used to summarize the strength of association between* H. pylori* seropositivity and risk factors in a multinomial logistic regression test. The level of statistical significance for the study was set at p<0.05. In all the tests, p-values less than 0.05 or near 0.05 were used as statistical association n for risk factors of* H. pylori* infection. The outcome measure was the detection of the presence of* H. pylori* antigens in stool.

## 3. Results

### 3.1. Demographic Characteristics of the Children and Parents/Guardians

A total of 304 children aged 1 to 15 years were recruited in the study of which 53.3% were girls (Table 1). The mean age of the children was 7 years with standard deviation of 4 years. Most of the children were 1 to 5 years old (42.8%) and 6 to 10 years old (37.5%) and the rest were older than 10 years. Majority of the children (79.6) were school going. More than half of the parents/guardians (53.0%) had at least university education with a majority (79.6%) living in permanent houses. Trade is the major activity of nearly half (49.3%) of the parents/guardians.

### 3.2. Seroprevalence of H. pylori Infection

Up to 96 of the participants were positive for* H. pylori* antibody test. When the 96 antibody positive children were requested for stool samples, only was 24.3% (74 of 304 children) had positive antigen test. Therefore, the overall prevalence of* H. pylori* infection was 24.3%.

**Figure 1:**
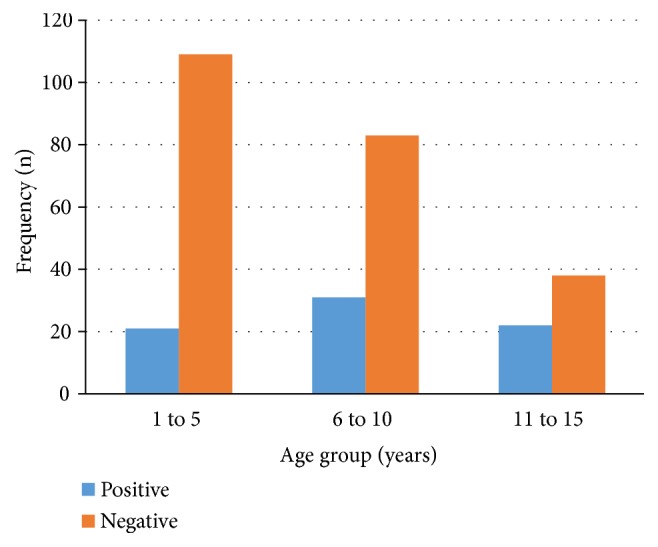
The distribution of* H. pylori* infection with age group (years) n=304.

The infection was highest among age group of 6 to 10 years (n=31, 41.9%) followed by the age group of 11 to 15 years (n=22, 29.7%) and lowest in the age group of 1 to 5 years (n=21, 28.4%) (Figure 1). The distribution of* H. pylori* seropositivity with gender was roughly similar to 38 (51.4%) of the cases in girls (p=0.2). Up to 68 (91.9%) of seropositive* H. pylori* results were from school going children compared to 6 (8.1%) in nonschool going children (p=0.003).

### 3.3. Factors Associated with H. pylori Infection

Several factors were studied about* H. pylori* seropositivity. The factors were gender, school attendance, sources of drinking water at schools and homes, the presence of hand washing sanitary facility at schools and homes, number of persons living in a home, family history of peptic ulcer disease and family history of stomach cancer (Tables 2 and 3). 36 out of 142 of the boys were* H. pylori* positive compared to 38 out of 124 of the girls (p=0.7). Majority of the infection occurred in children who attend schools compared to those who did not attend schools (68/174 vs 6/56, p=0.003). The infection rate was higher among children who had an unsafe source of water at their homes, had no sanitary facility at home, had overcrowded families or had an unsafe source of drinking water (p<0.001). Infection rate was seemingly similar among children who lacked sanitary facilities in their schools, had a family history of PUD or stomach cancer (p>0.001).

## 4. Discussion

The prevalence of the* H. pylori* infection was 24.3% among children aged 1 to 15 years among children attending Holy Innocents Hospital, Mbarara district. The prevalence in this study is low compared with findings from other studies among children in Kampala [19, 20] or different parts of Africa. However, our results are comparable to previous prevalence obtained in the neighbouring Kasese district where the prevalence of* H. pylori* was 29.9% [36]. The prevalence of* H. pylori* infection in other parts of Africa ranged between 40% and nearly 90% [37–41]. The low prevalence in our study would have been due to the recruitment of participants within a single or similar geographical characteristic. This would mean that some of the factors of transmission of* H. pylori* infection would be influenced by the environment [25, 31]. The observed low prevalence in our study could also be due to the rising usage of antibiotics such as Amoxicillin and Metronidazole in Uganda in the management of many infections like gastrointestinal disorders. This practice could have led to increased clearance of* H*.* pylori* and the resulting lowered prevalence. Being periurban, our study participants have a higher social class, with better socioeconomic standards to avoid known transmission sources such as contaminated water and foods [16].

It is also important to note that the antigen used in the serum antibody test was not from African or Ugandan origin and this might have affected the sensitivity of the test. The effect of the antigen difference, however, would have been small to affect the quality of the results.

The prevalence of* H. pylori* infection increased with increase in age from 16.2%, 27.2%, and 36.71% for children aged 1 to 5 years, 6 to 10 years, and 11 to 15 years, respectively. The trend is similar to what was demonstrated in others studies where infection rates increased with increase in age [10, 11, 15, 18, 28, 42–45]. The finding would suggest that colonization with* H. pylori* organism starts early in life. During neonatal life, sources of infections would be limited to person to person from caretakers, family members, or nursery attendants. As age increase, exposure to various infection sources increases hence the ultimate rate of infection. This finding can explain why the rate of infection is higher in school going children. The rate of infection can even be higher in children who attend schools with no or poor sanitary facilities and or lack of clean drinking waters. Early study in Bangladesh found out that the rate of infection with* H. pylori is* lower in children 1 to 3 months but steadily increased from 6 to 9 months and in older children [5].

The current study has shown no difference of* H. pylori* infection rate in both girls and boys (p=0.7, *χ*^2^, 0.15). This suggests that maintaining all exposures constant, both girls and boys would be infected equally [46]. In this study the association of school attendance as a risk factor for* H. pylori* infection compared to nonschool attendance. This suggests that, after commencing school, the poor hygiene at the schools increases the chance of infection with* H. pylori* bacteria. There is also concentration at schools increasing the chance of person-to-person transmission [47]. Person-to-person transmission can also occur at home as long as there is an infected person in the home for few months [48].

The study found a lower percentage of infection rate (4.9%) among children from households with 1 to 3 members compared to 19.4% among children from households with more than 3 members (p<0.001). This would still mean that overcrowding increases chances of acquiring* H. pylori* infection in children [11, 47].

Examining the multivariate model, school attendance, family size, family history of PUD, and the absence of hand washing facility increased the chance of acquiring* H. pylori* infection, shown by ODDS Ratio >1.0 (Table 3). Since school attendance seems to be confounded upon by other unmeasured or measured factors, control of* H. pylori* infection would be geared towards eliminating other transmission points among school going children. The abovementioned ways of eliminating* H. pylori* infection can include improving the sanitation at schools and at homes, early diagnoses and treatments of the infected, and general community sensitization [49–51]. This finding however disagreed with early finding from a study among Bangladeshi children where lack of sanitary facilities was not associated with* H. pylori* infection [52].

## 5. Conclusions

The prevalence of* H. pylori* among children presenting with gastrointestinal complaints at Holy Innocents Children's Hospital is high. Risk factors* H. pylori* among children presenting with gastrointestinal complaints at Holy Innocents Children's Hospital were school attendance, lack of sanitary facility at schools and homes, and having more than overcrowding at homes.

## Figures and Tables

**Table 1:** Demographic characteristics of the participants or parents/guardians (n=304).

Variable	Category	Frequency	Percentage
Sex	Female	162	53.3

Age group	1 to 5 years	130	42.8
	6 to 10 years	114	37.5
	11 to 15 years	60	19.7

District of residence	Mbarara	170	55.9
	Kiruhura	46	15.1
	Isingiro	57	18.8
	Others	31	10.2

School attendance	Yes	242	79

Source of drinking water at school	Tap water	164	53.9
	Borehole	37	12.2
	Spring	41	13.5
	Not applicable	62	20.4

Presence of a hand washing	Yes	194	63.8
facility and toilet at school	No	50	16.4
	Not applicable	60	19.7

Education level of parent/guardian	University	161	53.0
	Secondary	80	26.3
	Primary	48	15.8
	No formal education	15	4.9

**Table 2:** Risk factors for *H. pylori* infection.

variable		*H. pylori* result		Chi square test	P value
		Positive	Negative		
sex	Male	36	106	0.15	0.7
	Female	38	124		

School attendance	Yes	68	174	9.05	0.003
	No	6	56		

Source of school drinking water	Safe	58	205	5.55	0.02
	Unsafe	16	25		

Sanitary facility at school	Yes	59	197	1.48	0.2
	No	15	33		

Sanitary facility at home	Yes	52	209	19.56	<0.001
	No	22	21		

Family history of PUD	Yes	39	101	1.74	0.2
	No	35	129		

Family history of cancer of stomach	Yes	21	67	0.02	0.9
	No	53	163		

Source of home drinking water	Safe	46	182	8.6	0.003
	Unsafe	28	48		

Overcrowding in the family	Yes	59	122	16.6	<0.001
	No	15	108		

PUD: peptic ulcer disease.

**Table 3:** Multinomial logistic regression showing associations between *H. pylori* infection and risk factors.

Variable	Std. Err	Z score	Odds Ratio	95% CI	P value
Sex	0.338	0.48	1.151	0.647 – 2.045	0.633
School attendance	1.987	2.72	3.948	1.470 – 10.601	0.006
Source of school drinking water	0.423	-0.13	0.944	0.393 – 2.270	0.898
Sanitary facility at school	0.678	1.05	1.574	0.676 – 3.664	0.293
Source of drinking water at home	0.193	-1.70	0.549	0.275 – 1.094	0.089
Sanitary facility at home	0.089	-3.76	0.228	0.106 – 0.492	<0.001
Family history of PUD	0.570	1.72	1.751	0.925 – 3.314	0.085
Family history of Ca Stomach	0.263	-0.87	0.731	0.361 – 1.481	0.386
Family size	0.872	2.84	2.597	1.344 – 5.016	0.04

Std. Err: standard error; CI: confidence interval; PUD: peptic ulcer disease; Ca stomach: cancer of the stomach.

## Data Availability

The data is available on request from the corresponding author
